# Hypoglycemia incidence and behavioural adjustments during free‐living unstructured physical activity in adults with type 1 diabetes using AID systems: Results from the RAPPID study

**DOI:** 10.1111/dom.70122

**Published:** 2025-09-09

**Authors:** Michael Joubert, Laurent Meyer, Said Bekka, Luc Rakotoarisoa, Nicolas Scheyer, Bleuenn Dreves, Bruno Guerci

**Affiliations:** ^1^ Diabetes Care Unit Caen University Hospital, Unicaen Caen France; ^2^ Diabetes Care Unit Strasbourg University Hospital Strasbourg France; ^3^ Diabetes Care Unit Diabetes Center Mainvilliers France; ^4^ Diabetes Care Unit Nancy University Hospital Nancy France

**Keywords:** automated insulin delivery, continuous glucose monitoring, hypoglycemia, physical activity, type 1 diabetes

## Abstract

**Aims:**

To assess the frequency and management of hypoglycaemia during unstructured physical activity (PA) in adults with type 1 diabetes (T1D) using automated insulin delivery (AID) systems in real‐life settings.

**Materials and Methods:**

RAPPID is a prospective, multicenter, observational study conducted over 1 month in four French tertiary care centres. Adults with T1D using one of three AID systems (MiniMed 780G, Tandem t:slim X2 with Control‐IQ, or Ypsopump with CamAPS FX) and performing ≥2 unstructured PA sessions per week were included. Participants completed paper logbooks documenting each PA session (type, intensity, hypoglycemia, adjustments). Continuous glucose monitoring (CGM) and pump data were downloaded. Glycaemic control was assessed using CGM‐derived metrics within predefined peri‐exercise time windows.

**Results:**

Eighty‐six participants (mean age 42.5 ± 14.3 years; 43% women; diabetes duration 23.6 ± 13.1 years; mean HbA1c 52 ± 7 mmol/mol (6.9% ± 0.6%)) reported 954 PA sessions (73% aerobic; 61% moderate intensity). TBR (<70 mg/dL) increased from 1% pre‐exercise to 6% during and 5% post‐exercise (early recovery phase). Clinical hypoglycaemia occurred in 20% of sessions (one‐third of episodes were asymptomatic); 38% of participants experienced at least 1 level 2 event (<54 mg/dL). Anaerobic or high‐intensity sessions were associated with lower hypoglycaemia risk. Temporary targets were used in 73% of sessions but initiated ≥1 h before PA in only 27%. Carbohydrate intake before and during PA was frequent but often suboptimally timed or dosed.

**Conclusions:**

Hypoglycemia remains common during and after PA in AID users. Suboptimal adjustment strategies and impaired symptom awareness contribute to risk. Individualised education remains essential to enhance safety.

## 1 INTRODUCTION

Automated insulin delivery (AID) systems, which integrate an insulin pump, a continuous glucose monitor (CGM), and a control algorithm, are now considered the gold standard for the management of type 1 diabetes (T1D).^1^ They offer significant improvements in glycemic control and quality of life, without increasing the risk of hypoglycemia.^2^, ^3^, ^4^, ^5^, ^6^, ^7^ However, despite these technological advances, certain situations, such as meals and physical activity (PA), still require patient intervention, which can add a layer of complexity to diabetes management from thepatient's perspective. While PA is widely encouraged for individuals with type 1 diabetes,^8^, ^9^, ^10^, ^11^, ^12^, ^13^ its practice, especially in the context of aerobic exercise, remains a challenge due to the increased risk of hypoglycemia.^14^ As a result, the fear of glycemic imbalances remains one of the main barriers to regular PA practice among individuals with type 1 diabetes, and likely persists even among those using automated insulin delivery systems.^15^, ^16^ Indeed, for these individuals, managing PA can seem particularly complex, as it involves both adjustments of AID settings and appropriate dietary measures.

International guidelines for PA management with AID systems have been recently published.^17^ They highlight the numerous factors that influence the glycemic response to exercise, including the type, duration, and timing of activity, the amount of insulin on board, and glucose trends. In addition, these guidelines provide both general recommendations applicable to all systems and more specific adjustments—whether settings or dietary strategies—tailored to the characteristics of each individual system. Despite these guidelines, as well as data from several studies that have tested various system or dietary adjustments during standardised PA in clinical research settings,^18^, ^19^, ^20^, ^21^, ^22^ the management of exercise remains challenging for individuals with type 1 diabetes using AID systems. Moreover, data on unstructured PA performed in free‐living conditions are still very limited, and the applicability of these new recommendations under everyday circumstances remains to be evaluated.

The RAPPID study aimed to assess the frequency and management of hypoglycemia associated with unstructured PA in real‐life conditions in people with type 1 diabetes using AID systems.

## 2 MATERIALS AND METHODS

### 2.1 Study design

This was a non‐interventional, longitudinal, prospective, multicenter, real‐life French study conducted with four specialised tertiary diabetes care centers (Vandœuvre‐lès‐Nancy, Strasbourg, Caen, and Mainvilliers) among TD1 patients using an AID, with a 1‐month follow‐up. The design, implementation, and interpretation of the study were overseen by a Scientific Committee of four diabetes specialists. The study protocol was approved by Ethic Committee Ouest II, Angers, France, and registered with the French National Agency for the Safety of Medicines and Health Products. It was registered on clinicaltrial.gov with the number NCT07015970. A non‐opposition to participate in the study was obtained from all participants, in accordance with the simplified French accessibility regulation MR‐003.

The primary objective of this study was to characterise glycemic control in physically active adults with type 1 diabetes by assessing time below range (TBR) at thresholds of <70 mg/dL and <54 mg/dL across five predefined peri‐exercise time periods (see below). Secondary objectives included describing time in range (TIR, 70–180 mg/dL), time above range (TAR, >180 mg/dL and >250 mg/dL), the occurrence and symptoms related to hypoglycemia as reported by participants and the use of device‐based and dietary adjustment strategies to mitigate hypoglycemia risk.

### 2.2 Participants

Participants were adults T1D patients using for at least 3 months one of the three AID systems available in France at the time of the study: Minimed 780G (Medtronic) with Guardian 4 sensor, or T:Slim X2 (Tandem) with Dexcom G6 sensor and CONTROL IQ algorithm, or Mylife Ypsopump (Ypsomed) with Dexcom G6 sensor and CamAPS FX application, engaging in regular PA as part of daily life (≥2 unstructured PA sessions per week, each lasting more than 30 min) (Supplementary Figure 1). Additional inclusion criteria were to be able to download data from the pump and to complete the PA log and self‐questionnaires. Subjects already participating in a clinical trial, pregnant or breastfeeding women (or planning a pregnancy during the study), adults under legal protection (guardianship, curatorship, or judicial safeguard), patients with decompensated retinopathy or cardiac disease, patients with a history of severe hypoglycaemia during PA in the past 6 months, and patients with unresolved diabetic foot trophic disorders were excluded from the study.

All participants received standardised guidance on the management of their AID system and dietary adjustments, consistently delivered across the four participating centres. These recommendations represented a pragmatic and simplified adaptation of the international consensus on AID use available at the time of the study^23^ and were intended to optimise glycemic control during PA in real‐life conditions (Supplemental Table 1). System‐related adjustments included activating a higher glucose target (temporary target) 1–2 h before each planned PA, for a duration covering the session. In case of persistent post‐exercise hypoglycemia risk, maintaining the temporary target for 2–8 h after activity was advised. Dietary instructions emphasised accurate carbohydrate announcement before exercise, especially when no temporary target had been set before the meal bolus. Participants were instructed to avoid unannounced snacks in the hour before PA and to limit carbohydrate intake during exercise to ≤20 g at a time. These measures aimed to prevent algorithmic counterregulation and excessive active insulin, thereby reducing hypoglycemia risk. Recommendations were reiterated, but participants were encouraged to adapt them based on personal glycemic responses to exercise.

Participants were enrolled between March and October 2024; last patient follow‐up ended November 2024.

### 2.3 Procedure

Two physician‐led data collection time points were planned. The first occurred at baseline and included demographic and clinical characteristics, glycemic control within the month preceding inclusion (e.g., occurrence of diabetic ketoacidosis or hypoglycemia, HbA1c), diabetes management data (duration of AID system use, type of insulin therapy), and information on usual PA, including session duration, type (aerobic, anaerobic, or mixed), sport practiced, competition participation, and adherence to a specific diet. At baseline, participants also completed validated questionnaires assessing quality of life (Audit of Diabetes‐Dependent Quality of Life—ADDQoL),^24^ treatment satisfaction (Diabetes Treatment Satisfaction Questionnaire—DTSQs),^25^ and fear of hypoglycemia (Hypoglycemia Fear Survey II—HFS‐II).^26^ These questionnaires are presented in the supplementadl method.

The second data collection point took place at the 1‐month follow‐up and involved downloading insulin pump and glucose sensor data. This download covered a period spanning from 15 days prior to the first reported PA session to 15 days after the last session.

Throughout the 1‐month study period, participants completed a paper logbook after each unstructured PA session, documenting session timestamp, type of activity, self‐reported intensity (using a modified Borg scale), duration, any AID system adjustments, dietary strategies, occurrence of hypoglycaemia and associated symptoms, snacks consumed, and treatments for hypoglycaemia. For each reported session, investigators subsequently reviewed the description of the activity and validated or corrected its categorisation into aerobic, anaerobic, or mixed. In cases of disagreement, a collegial discussion was held, and consensus was reached before final classification.

### 2.4 Statistical analyses

Based on feasibility over a 6‐month inclusion period and the descriptive aim of the study, a sample size of 30 patients per group was determined. This was derived from the expected precision of the primary outcome (TBR), assuming a standard deviation of up to 13% and allowing for 15% early discontinuation. The target was compatible with the recruitment capacities of the four participating centres.

Statistical analyses were conducted using SAS software (version 9.4; SAS Institute, Cary, NC). A two‐sided type I error rate (*α*) of 0.05 was used for all analyses. Descriptive statistics were reported as counts and percentages for categorical variables, and as means ± standard deviations (SD) for continuous variables, unless otherwise specified. Missing data are indicated when relevant (*n*, %).

The primary outcome was the percentage of time spent below range (TBR) as measured by CGM, defined as <70 mg/dL (level 1) and <54 mg/dL (level 2). Secondary outcomes included TIR (70–180 mg/dL) and TAR, categorised as >180 mg/dL (level 1) and >250 mg/dL (level 2). These glycemic metrics were analysed across five predefined time windows: pre‐exercise (−2 h), during exercise, early recovery (+3 h post‐exercise), late recovery (from 3 h post‐exercise to 8:00 a.m. the following day or until the next pre‐exercise window if sessions were closely spaced), and non‐exercise periods (all remaining time not covered by the preceding windows).

Hypoglycemic episodes reported by participants in their logbooks (based on symptoms and/or observed low glucose values), adjustments to the system settings (primarily the use of a temporary target), and dietary behaviours (carbohydrate intake around exercise sessions) were descriptively analysed across periods before, during, and after PA. These time windows, derived from patient‐reported data, differed from the standardised CGM‐based periods used for glucose analyses. In addition, mixed‐effects logistic regression models were performed to assess the association between deviations from recommendations and the risk of reported hypoglycemia. All analyses were conducted overall and stratified when relevant by pump type, PA type, PA intensity, and self‐reported treatment adjustment practices.

## 3 RESULTS

### 3.1 Participant characteristics

Of 87 participants, one was excluded for reporting only a single PA session during follow‐up. Thus, 86 participants were included in the final analysis (Supplementary Figure 1). Overall, 57% were male, the mean age was 42.5 ± 14.3 years, 80% were professionally active, and 78% had higher education. The mean diabetes duration was 23.6 ± 13.1 years, and AID system use averaged 18.3 ± 11.7 months. Diabetes‐related complications were present in 19% (mainly retinopathy, 11%). Impaired hypoglycemia awareness (Gold score ≥4) was noted in 7%, and 17% reported at least one asymptomatic episode in the preceding month. The mean HbA1c was 52 ± 7 mmol/mol (6.9% ± 0.6%). One participant experienced severe hypoglycemia and one a ketoacidosis event before inclusion. Detailed characteristics are presented in Table 1.

**TABLE 1:** Demographic and descriptive baseline characteristics of the participants, overall and according to the treatment used.

	Overall	Medtronic	Tandem	Ypsomed
Baseline characteristics	*n* = 86	*n* = 39	*n* = 25	*n* = 22
Gender, *n* (%)				
Female	37 (43)	17 (44)	12 (48)	8 (36)
Male	49 (57)	22 (56)	13 (52)	14 (64)
Age, mean ± SD (years)	42.5 ± 14.3	44.8 ± 16.3	39.8 ± 13.6	41.4 ± 11.0
Employment, *n* (%)				
Active	69 (80)	28 (72)	22 (88)	19 (86)
Retired	10 (12)	8 (20)	2 (8)	0 (0)
Student	7 (8)	3 (8)	1 (4)	3 (14)
Most recent occupation, *n* (%)				
Managers and higher intellectual professions	37 (43)	14 (36)	13 (52)	10 (46)
Employee	26 (30)	16 (41)	6 (24)	4 (18)
Intermediate professions	7 (8)	2 (5)	3 (12)	2 (9)
Craftsman, merchant, and business owner	5 (6)	3 (8)	0 (0)	2 (9)
Worker	5 (6)	1 (2)	2 (8)	2 (9)
Others	6 (7)	3 (8)	1 (4)	2 (9)
Level of education, *n* (%)				
Higher education	67 (78)	27 (69)	21 (84)	19 (86)
High school level	15 (17)	10 (26)	2 (8)	3 (14)
Primary/Middle school	4 (5)	2 (5)	2 (8)	0 (0)
BMI, mean ± SD (kg/m^2^)	25.4 ± 3.5	24.7 ± 3.0	25.8 ± 3.9	26.1 ± 3.6
Smoking status, *n* (%)				
Non‐smoker	60 (70)	27 (69)	15 (60)	18 (82)
Ex‐smoker	21 (24)	12 (31)	7 (28)	2 (9)
Current smoker	5 (6)	0 (0)	3 (12)	2 (9)
Diabetes duration, mean ± SD (years)	23.6 ± 13.1	26.3 ± 13.6	21.3 ± 12.5	21.3 ± 12.7
Duration of AID, mean ± SD (months)	18.3 ± 11.7	19.7 ± 11.7	19.6 ± 9.2	14.2 ± 13.6
Insulin type, *n* (%)				
Aspart	57 (66)	22 (56)	16 (64)	19 (86)
Lispro	28 (33)	16 (41)	9 (36)	3 (14)
Glulisine	1 (1)	1 (3)	0 (0)	0 (0)
Daily insulin dose, mean ± SD (UI/day)	45.9 ± 18.1	43.9 ± 16.8	50.8 ± 22.7	44.3 ± 14.2
Diabetes complications and comorbidities type, *n* (%)				
Diabetic retinopathy	9 (11)	5 (13)	3 (12)	1 (4.5)
Diabetic neuropathy/Diabetic foot	2 (2)	1 (3)	1 (4)	0 (0)
Diabetic nephropathy	1 (1)	0 (0)	1 (4)	0 (0)
Coronary artery disease	2 (2)	2 (5)	0 (0)	0 (0)
Peripheral artery disease	2 (2)	2 (5)	0 (0)	0 (0)
Heart failure	1 (1)	1 (3)	0 (0)	0 (0)
Gold score ≥4	6 (7)	0 (0)	4 (16)	2 (9)
During the prior month				
Ketoacidosis, *n* (%)	1 (1)	0 (0)	0 (0)	1 (5)
Severe hypoglycemia, *n* (%)	1 (1)	0 (0)	0 (0)	1 (5)
Moderate and symptomatic hypoglycemia, *n* (%)	64 (74)	27 (69)	18 (72)	19 (86)
Asymptomatic hypoglycemia, n (%)	15 (17)	4 (10)	8 (32)	3 (14)
HbA1c available during the prior month, *n* (%)				
Mean ± SD (mmol/mol)	52 ± 7	53 ± 7	50 ± 7	52 ± 7
Mean ± SD (%)	6.9 ± 0.6	7.0 ± 0.6	6.7 ± 0.6	6.9 ± 0.6
Missing data, *n* (% of total)	9 (10)	5 (13)	3 (12)	1 (5)
Usual physical activity profile				
Competitive athlete, *n* (%)	15 (20.5)	10 (27.8)	2 (9.5)	3 (18.8)
Monthly physical activity sessions (baseline), *n* (%)				
1–5 sessions	4 (5.5)	3 (8.3)	1 (4.8)	0
6–10 sessions	38 (52.1)	16 (44.4)	9 (42.9)	13 (81.3)
11–15 sessions	10 (13.7)	5 (13.9)	4 (19.0)	1 (6.3)
16–20 sessions	16 (21.9)	10 (27.8)	4 (19.0)	2 (12.5)
20+ sessions	5 (6.8)	2 (5.6)	3 (14.3)	0

At baseline, participants reported good quality of life (QoL score: 1.3 ± 0.7), moderate diabetes impact (ADDQoL: −1.8 ± 1.4), high treatment satisfaction (DTSQs: 30.1 ± 3.7), and low hypoglycemia fear scores (behaviour: 6.8 ± 3.2; anxiety: 5.3 ± 3.2) (Supplementary Table 2).

### 3.2 PA sessions

A total of 954 PA sessions were reported (Supplementary Figure 1), with a mean duration of 1.5 ± 1.1 h. Most were aerobic (73%) and rated low (27%) or moderate (61%) intensity. Endurance sports were most common (73%), followed by fitness/gym activities (16%). Session characteristics appear in Table 2.

**TABLE 2:** Characteristics of physical activity sessions declared by participants in the paper logbook overall and according to AID system.

	Overall	Medtronic	Tandem	Ypsomed
Physical activity characteristics	*n* = 954	*n* = 514	*n* = 270	*n* = 170
PA session duration				
Mean ± SD (hours)	1.5 ± 1.1	1.6 ± 1.2	1.5 ± 1.0	1.3 ± 0.8
Missing data, *n* (%)	9 (1)	4 (1)	4 (2)	1 (1)
Type of PA sessions, *n* (%)				
Aerobic	692 (73)	374 (73)	204 (75)	114 (67)
Anaerobic	85 (9)	44 (8)	29 (11)	12 (7)
Mixed	144 (15)	80 (16)	23 (9)	41 (24)
Missing data	33 (3)	16 (3)	14 (5)	3 (2)
Modified Borg scale intensity of PA sessions, *n* (%)				
Nothing (0)	16 (2)	10 (2)	6 (2)	0 (0)
Low (0.5–2)	257 (27)	157 (31)	48 (18)	52 (31)
Moderate (3–5)	584 (61)	316 (61)	168 (62)	100 (59)
High (6–10)	88 (9)	25 (5)	47 (17)	16 (9)
Missing data	9 (1)	6 (1)	1 (<0.1)	2 (1)
WHO effort scale intensity of PA sessions, *n* (%)				
Mild	320 (34)	168 (33)	82 (30)	70 (41)
Moderate	431 (45)	246 (48)	115 (43)	70 (41)
High	171 (18)	83 (16)	62 (23)	26 (16)
Very high	13 (1)	1 (<1)	10 (4)	2 (1)
Missing data	19 (2)	16 (3)	1 (<1)	2 (1)
Type of sport practiced during PA sessions, *n* (%)				
Endurance sports	696 (73)	373 (73)	198 (73)	125 (74)
Fitness/Gym	152 (16)	79 (15)	42 (16)	31 (18)
Swimming	26 (3)	6 (1)	15 (6)	5 (<1)
Racket sports	24 (2)	19 (4)	4 (1)	1 (<1)
Domestic activities (gardening, DIY)	14 (1)	4 (<1)	7 (2)	3 (<1)
Agility sports (archery, equestrianism)	9 (<1)	8 (2)	1 (<1)	0 (0)
Combat sports	6 (<1)	6 (1)	0 (0)	0 (0)
Team ball sports	5 (<1)	4 (<1)	1 (<1)	0 (0)
Karting	1 (<1)	1 (<1)	0 (0)	0 (0)
Missing data	21 (2)	14 (3)	2 (<1)	5 (<1)

### 3.3 Glucose control and hypoglycemia occurrence

**FIGURE 1:**
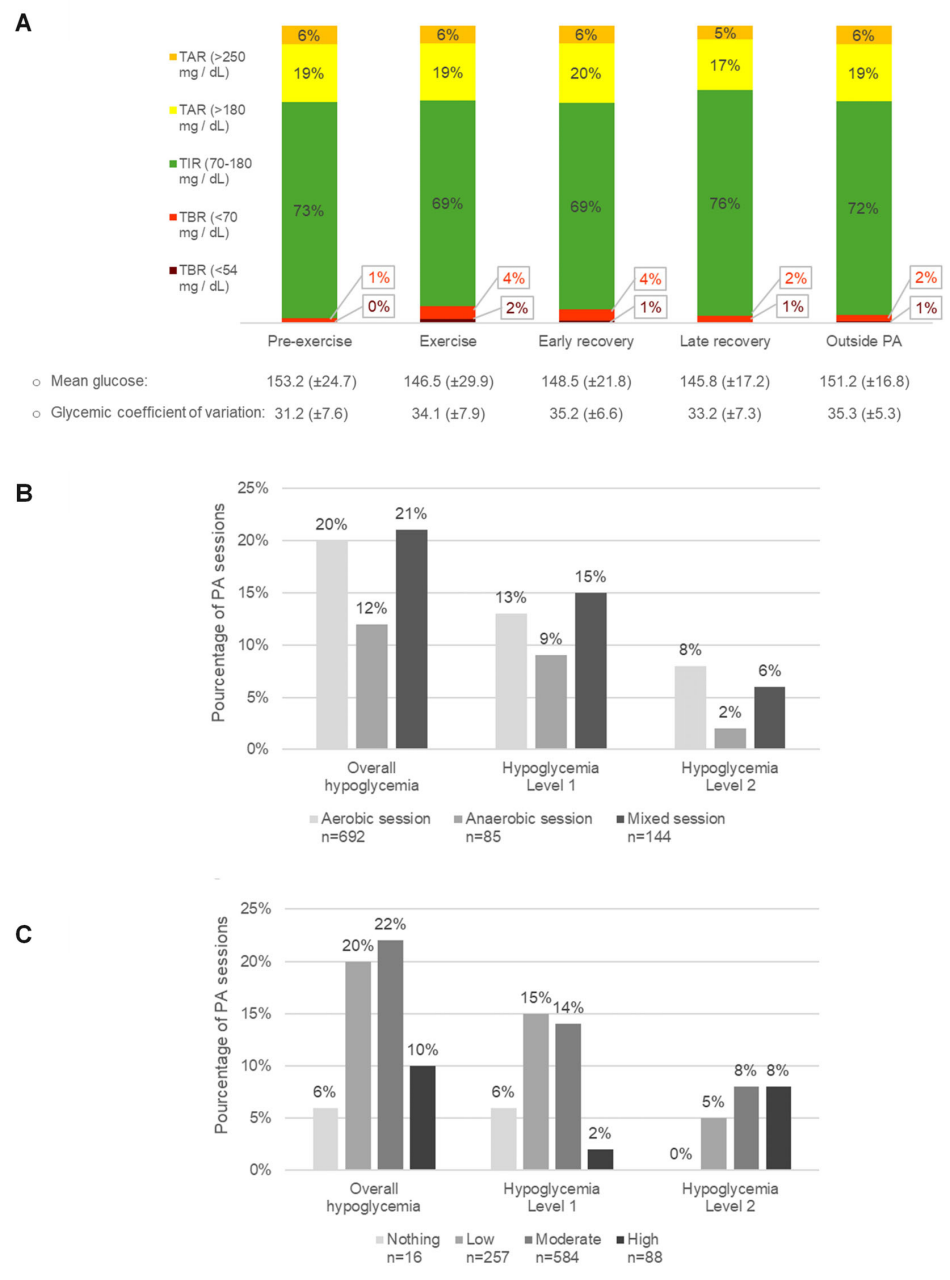
Continuous glucose monitoring (CGM) metrics (A) (*n* = 714) across predefined time windows: Pre‐exercise (−2 h), during exercise, early recovery (+3 h post‐exercise), late recovery (from 3 h post‐exercise to 8:00 a.m. the following day or until the start of the next pre‐exercise window when sessions were closely spaced), and non‐exercise periods. Frequency of patient‐reported hypoglycaemia episodes according to the type of physical activity session (aerobic, anaerobic, or mixed) (B), and according to perceived intensity based on the modified Borg scale (C) (*n* = 954).

Primary and main secondary outcomes derived from CGM data are shown in Figure 1A and concern the subset of 714 PA sessions (out of 954) for which CGM data were available. During ‘non‐exercise’ periods, which accounted for the vast majority of the one‐month study timeframe, overall TBR was 3% (TBR <70: 2%, TBR <54: 1%) and TIR (70–180 mg/dL) was 73%, reflecting the participants' baseline glycemic control. TBR increased to 6% during exercise and remained elevated at 5% during the early recovery phase, and 4% during the late recovery phase, compared to 1% in the pre‐exercise period. TAR remained stable across the different periods (25%–26%), except during late recovery, where it decreased to 22%, coinciding with an increase in TIR from 69% (during and immediately after exercise) to 76%.

From logbooks, 78% of participants reported at least one hypoglycemia episode; 22% reported none. Level 2 hypoglycemia affected 38% of participants, impacting about 20% of sessions (Supplementary Table 3). Among all sessions, 191 (20%) included hypoglycemia, two‐thirds being level 1. Symptoms were reported in 65% of these sessions—most often combined adrenergic and neuroglycopenic signs (42%) (Supplementary Table 4). About one‐third of episodes were asymptomatic, with a recognition of hypoglycemia based on CGM or capillary readings. Activity was temporarily interrupted in 19% and permanently in 12% of hypoglycemic sessions. Aerobic and mixed sessions were more frequently associated with hypoglycemia (20% and 21%) than anaerobic (12%). Level 1 events occurred more often in low/moderate intensity sessions (15%/14%), while level 2 events were more frequent in moderate/high‐intensity sessions (8% each). Distributions of hypoglycemic episodes, according to both exercise type and intensity, are shown in Figure 1B,C.

### 3.4 Adjustment practices

Among 824 sessions with adjustment data, a temporary target was used in 73%, but set over 1 h before in only 27%. In 46%, it was activated less than an hour before or during PA; 27% had no activation. Carbohydrate intake was reported in 36% of sessions within the 2 h preceding PA, with a median amount of 20 g [IQR 15–30]. During exercise, carbohydrate intake was documented in 27% of sessions, with a median of 25.5 g [IQR 18–45]; an intake exceeding 20 g at once occurred in 10% of sessions. These adjustment behaviours are summarised in Table 3. None of these deviations, whether isolated or combined, were significantly associated with an increased risk of hypoglycemia (Supplementary Table 5).

**TABLE 3:** Adjustment practices performed for PA sessions, overall and according to AID system.

Adjustment practices	Overall	Medtronic	Tandem	Ypsomed
Temporary target, *n* (%)	*n* = 824	*n* = 430	*n* = 226	*n* = 168
Activation at least 1 h before PA session	224 (27)	123 (29)	66 (29)	36 (22)
Activation less than 1 h or during PA session	336 (46)	221 (51)	76 (34)	83 (49)
No activation	219 (27)	86 (20)	84 (37)	49 (29)
Carbohydrate intake before (−2 h) PA	*n* = 954	*n* = 514	*n* = 270	*n* = 170
Number of PA sessions, *n* (%)	342 (36)	172 (33)	116 (43)	54 (32)
Carb amount (g), median [IQR]	20.0 [15.0;30.0]	20.0 [15.0;30.0]	20.0 [15.0;30.0]	20.0 [15.0;30.0]
Carbohydrate intake during PA				
Number of PA sessions, *n* (%)	257 (27)	126 (25)	90 (33)	41 (24)
Carb amount (g) median [IQR]	25.5 [18.0;45.0]	25.0 [18.0;40.0]	32.0 [21.0;54.0]	20.0 [12.0;28.0]
% PA sessions with carb intake >20 g at a time	10	8	18	4

## 4 DISCUSSION

Our study showed that, despite the use of advanced AID systems, hypoglycemia remains frequent during and after unstructured free‐living PA in individuals with type 1 diabetes, occurring in about one out of every four sessions, with nearly 40% of participants experiencing at least one level 2 hypoglycemic event during the 1‐month PA protocol period. CGM data confirmed this risk, with TBR (<70 mg/dL) increasing from 1% in the 2 h preceding exercise to 6% during activity and remaining elevated at 5% in the early recovery phase. Notably, in 31% of cases, these hypoglycemic events led to a temporary or permanent interruption of the exercise session, further highlighting their impact on the feasibility and safety of PA in daily life.

These results echo findings from the T1DEXI study, which demonstrated a significant hypoglycaemia risk during PA across insulin modalities.^27^ Involving 497 adults with T1D, T1DEXI included six structured aerobic, interval, or resistance sessions. CGM showed consistent glucose declines: −18 ± 39 mg/dL for aerobic, −14 ± 32 for interval, and −9 ± 36 for resistance training. Among AID users, aerobic sessions caused a −19 mg/dL drop. This confirms that even with automated systems, exercise remains a physiological challenge.

In RAPPID, hypoglycaemia varied by activity type: it was less frequent during anaerobic sessions than aerobic or mixed ones (Figure 1B). This aligns with known physiology: aerobic activity consumes glucose and lowers levels in the presence of exogenous insulin, while anaerobic efforts (e.g., sprinting, weightlifting) trigger counter‐regulatory hormones that may increase glucose.^11^ Yet, patients often struggle to classify activities metabolically. Perceived intensity, such as via the Borg scale, may offer a more intuitive risk predictor. In our data, sessions rated low or moderate intensity (often aerobic or mixed) were more associated with hypoglycaemia than high‐intensity (typically anaerobic) sessions, reinforcing this approach.

Regarding the perception of hypoglycemic episodes, only 7% of participants had a Gold score ≥4, the conventional threshold for impaired awareness, and 17% had reported at least one asymptomatic episode in the month preceding the study. Compared to the Hypo‐METRICS study, in which nearly one‐quarter of individuals with type 1 diabetes had impaired awareness (Gold score ≥4),^28^ these findings suggest that our physically active cohort may include individuals particularly experienced and vigilant in detecting hypoglycemia. However, approximately one‐third of hypoglycemic events during exercise were asymptomatic, raising concerns about a transient attenuation of awareness in this specific context. This suggests that PA itself may impair hypoglycemia perception, independently of baseline awareness status. To our knowledge, this is the first real‐life study to suggest such activity‐induced unawareness, although it is well established from physiological studies that moderate‐intensity exercise can blunt autonomic responses to hypoglycemia.^29^ Experimental studies, although scarce, provide additional support for this concept. In an animal model, a single bout of prolonged exercise markedly attenuated counterregulatory responses to insulin‐induced hypoglycemia performed immediately afterwards.^30^ In humans with type 1 diabetes, two prolonged bouts of low‐to‐moderate intensity exercise were shown to blunt autonomic, metabolic, and symptomatic responses to a standardised hypoglycemic clamp on the following day.^31^ Together, these findings highlight that exercise can acutely and persistently reduce counterregulatory capacity, thereby predisposing to transient hypoglycemia unawareness in the peri‐exercise period. Moreover, exercise‐related symptoms such as sweating, tachycardia, and sympathetic activation may mask adrenergic signs, making hypoglycemia harder to recognise. Supporting this, adrenergic signs were reported in isolation in only 15% of symptomatic cases. This blunted perception may delay detection and treatment, increasing the risk of deeper glycemic drops and level 2 hypoglycemia. Notably, the right panel of Figure 1C shows that level 2 episodes were more frequent during sessions perceived as moderate to intense, when sympathetic activation is likely maximal, supporting the hypothesis that adrenergic symptoms may be less easily identified under these conditions. Although this mechanism could theoretically favour severe hypoglycemia, no such events occurred in our cohort.

Our findings also underscore how suboptimal implementation of adjustment strategies contributes to this persistent risk. A temporary target was used in 73% of sessions, but only 27% were set over 1 h before exercise—the optimal window to reduce active insulin. In T1DEXI, pre‐exercise insulin reduction was applied in just 20% of cases.^32^ Timing matters: in MiniMed 780G users, setting the Temp Target 90 min before exercise along with a 25% bolus reduction was more effective than shorter lead times.^19^ Similarly, MiniMed 670G users reached 100% time‐in‐range during PA when the temporary target was activated 2 h before.^33^ A randomised crossover study with a model predictive AID system also showed that adjusting glucose targets and reducing meal bolus 60 or 120 min prior to moderate aerobic exercise prevented hypoglycemia equally well.^18^ Together, these findings validate current guidelines recommending Temp Target activation 1–2 hours pre‐exercise.^17^ Potential barriers to the timely implementation of temporary targets in real‐life conditions may include forgetfulness, limited knowledge of optimal timing, usability constraints of the devices or overreliance on AID systems. This underlines the importance of tailored educational support to help patients integrate these adjustments more systematically into their exercise routine.

Nutritional adjustments were also inconsistently applied. Pre‐exercise snacks were reported in 36% of sessions (median 20 g), often large or untimed. While likely intended as prevention, such behaviour may have triggered algorithm‐driven overcorrection and excess insulin delivery, increasing hypoglycaemia risk. This contradicts guidance advising against carbohydrate intake when glucose is within or above range due to rebound risk.^23^ Conversely, in situations where carbohydrate intake is clearly indicated—such as low glucose levels before exercise—adherence is equally poor: in T1DEXI, only 4% of users followed this recommendation.^32^ Both studies highlight a dual issue: unnecessary intake when not indicated and insufficient intake when it is.

Intra‐exercise carbohydrate intake was reported in only 27% of RAPPID sessions, despite high hypoglycaemia rates. The 2025 EASD/ISPAD consensus recommends 3–20 g fast‐acting carbs every 20–30 minutes when glucose is falling and below 6.7 mmol/L, guided by CGM arrows.^17^ Limited adherence may have contributed to poor glucose control. Uniform recommendations may not suit diverse activities, underscoring the need to help patients personalise carbohydrate strategies by context and preference.

Surprisingly, no significant association was observed between adherence to adjustment or dietary recommendations and hypoglycemia risk. This finding likely reflects the real‐world context of unstructured, self‐directed exercise, where participants applied one or several recommendations at their own discretion. A more constrained methodology, involving standardised exercise protocols and stepwise implementation of specific recommendations, would be required to clarify their relative contribution to dysglycemia prevention.

Altogether, these results highlight the urgent need for reinforced education to support patients transitioning to AID systems. Many individuals in the RAPPID cohort had previously used conventional insulin pumps, where dietary strategies around exercise, particularly carbohydrate intake, could be more flexible and often relied on empirical, patient‐led adjustments. With AID systems, however, these habits may inadvertently provoke algorithm‐driven overcorrections, underscoring the need to rethink and reframe established behaviours. Users need to understand how anticipatory adjustments (namely precise carbohydrate intake and timely activation of a temporary glucose target) interact with automated insulin delivery to optimise exercise safety and glycaemic outcomes in this new technological context. Future approaches should focus on facilitating the implementation of guideline‐recommended strategies in real‐life conditions. This could include educational interventions tailored to exercise routines, integration of reminders within AID devices, or simplified algorithms helping users anticipate and adjust in a timely manner.

Several limitations must be acknowledged for our study. First, the observational design precludes any causal inference regarding the impact of specific adjustment strategies on glycemic outcomes. Second, the use of self‐reported paper logbooks may have introduced recall bias or underreporting of dietary adjustments and hypoglycemia symptoms, although participants were instructed to complete the logbook entries in real time as closely as possible to the activity sessions. Third, it would have been valuable to equip participants with wearable devices (such as actimeters and heart rate monitors) during PA sessions, as these data could have enriched the analysis of exercise intensity, energy expenditure, and physiological responses, and could also have helped to categorise the type of PA more objectively. Fourth, data on insulin‐on‐board were not available, which precludes assessment of its potential influence on hypoglycemia risk during and after PA. Fifth, while the study included a substantial number of sessions, the follow‐up period was limited to 1 month, which may not capture long‐term behavioural adaptation or seasonal variations in activity patterns. Future studies with longer follow‐up and integration of wearable activity monitors could further refine our understanding of AID management during exercise in daily life. Finally, we did not attempt to compare outcomes between devices, as such analyses would not be meaningful given the limited sample size, the non‐randomised design, the lack of standardised PA sessions, and the different strategies and recommendations available to manage PA across systems.

However, taken together, the findings of the RAPPID study provide valuable insights into the real‐world challenges of managing PA in adults with type 1 diabetes using advanced AID systems. Despite the technological sophistication of these devices, hypoglycaemia remains frequent during and after exercise, particularly in the context of suboptimal adjustment strategies. The study also highlights the potential utility of perceived exertion scales as practical tools for patients to self‐assess hypoglycaemia risk, particularly given the limitations of metabolic classification in diverse sporting contexts. Furthermore, the high proportion of asymptomatic hypoglycaemia observed during exercise raises important concerns regarding activity‐induced impairment in hypoglycaemia awareness, which may not be captured by conventional baseline screening tools.

Importantly, RAPPID is the first prospective, multicentre, real‐life study to integrate behavioural, glycemic, and technological data across a broad range of physical activities in adults using AID systems. Its comprehensive approach and large dataset strengthen the external validity of the findings and offer a unique perspective on the implementation of international exercise guidelines in routine care. These results emphasise the need for enhanced education and structured support for individuals with type 1 diabetes engaging in PA, to bridge the gap between technological capabilities and real‐world safety. As AID systems become increasingly widespread, tailored strategies addressing the specific demands of exercise will be essential to fully realise their potential in improving both glycemic control and quality of life. In addition, future generations of AID systems will likely need to integrate additional sensors (e.g., for meal or activity detection), implement improved algorithms that account for insulin on board at the onset of PA, and leverage individualised behavioural patterns to anticipate disturbances and further enhance real‐world outcomes.

## AUTHOR CONTRIBUTIONS

M.J., L.M., S.B., and B.G. contributed to study design, participant recruitment, data collection, interpretation of data, and reviewed and edited the manuscript. L.R., N.S., and B.D. reviewed and edited the manuscript. M.J. drafted the manuscript and coordinated revisions. All authors approved the final version of the manuscript. B.G. is the guarantor of this work and, as such, had full access to all the data in the study and takes responsibility for the integrity of the data and the accuracy of the data analysis.

## FUNDING INFORMATION

This study was supported by an unrestricted educational grant from ISIS Diabète.

Study coordination, data monitoring, statistical analysis and methods/results drafting were performed by Qualees, which was contracted by ISIS Diabète. ISIS Diabète and Qualees had no role in data interpretation nor publication decisions and operated under the supervision of the academic investigators.

## CONFLICT OF INTEREST STATEMENT

M.J. declares consultant and/or speaker fees and/or research support from Abbott, Amgen, Astrazeneca, Boehringer‐Ingelheim, Dexcom, Glooko, Insulet, ISIS Diabète, Lifescan, Lilly, Medtronic, Novonordisk, Qualees, Roche Diabetes, Sanofi, Tandem, Ypsomed. L.M. declares consultant and/or speaker fees and/or research support from Abbott, Astra‐Zeneca, Boehringer‐Ingelheim, Dexcom, Glooko, ISIS Diabète, Lilly, Medtronic, NovoNordisk, Qualees, Sanofi, MSD, Dinno Santé. S.B. declares consultant and/or speaker fees and/or research support from AstraZeneca, BMS, Abbott, MSD, Isis Diabète, Medtrum, Novo, Sanofi, Takeda, Lifescan, MSD, Novartis, Medtronic, Johnson, Urgo, Lilly, Boehringer‐Ingelheim. L.R. declares consultant and/or speaker fees and/or research support from Lilly. N.S. declares consultant and/or speaker fees and/or research support from Ascendis Pharma, Esteve, Ipsen, Lilly, Merck, Novonordisk, Pfizer, and Recordati Rare Disease. B.D. declares consultant and/or speaker fees and/or research support from Ascendis Pharma, Gilead, Lilly, Sanofi, Abbott, Novonordisk, and Boehringer‐Ingelheim. B.G. declares consulting, expert opinions, writing, and proofreading work, participation in phase II, III, and IV clinical studies, speakers at symposia, co‐funding or grants for clinical research projects for the following pharmaceutical and industrial companies, healthcare providers, official organizations, and scientific societies: Novo Nordisk, Eli Lilly, Johnson and Johnson, AstraZeneca, Boehringer Ingelheim, Bristol‐Myers Squibb, Bayer, Sanofi Aventis, GlaxoSmithKline, Novartis, Janssen, Intarcia, Metacure, Insulet, Pfizer, MSD, Roche Diagnostic, Medtronic, Menarini Diagnostic, Ypsomed, Abbott, Dexcom, Lifescan, Vitalaire, Dinno Santé, Ork'yn, Asten, ISIS diabète, CEMKA, SANOIA, SEMEIA, AFSSAPS, CNAMTS, CEPS, ANSM, CNEDIMTS, GMED, EASD, SFD, SFE, NSFA, EASD (EUDF).

## PEER REVIEW

The peer review history for this article is available at https://www.webofscience.com/api/gateway/wos/peer‐review/10.1111/dom.70122.

## Supporting information


**Data S1:** Supporting Information

## Data Availability

The data that support the findings of this study are not openly available but are available from the corresponding author upon reasonable request.
