# Supplementary material for: Hypoglycemia incidence and behavioural adjustments during free‐living unstructured physical activity in adults with type 1 diabetes using AID systems: Results from the RAPPID study
Source: Diabetes Obes Metab. 2025 Sep 9;27(12):7221–31. doi: 10.1111/dom.70122 (PMC12587226; doi:10.1111/dom.70122)
Supplement: Supplementary file 1 — Data S1: Supporting Information [file DOM-27-7221-s001.docx]

Supplementary table 1 - Practical recommendations delivered to patients for AID use during physical activity

- Explain to patients how to identify activities with higher hypoglycemia risk, typically aerobic or endurance-type exercise (e.g., running, cycling, swimming, team sports)
- Activate higher glucose target (temporary target) 1–2 h before exercise and maintain throughout the session
- If post-exercise hypoglycemia risk persists, extend temporary target 2–8 h after activity
- Avoid unannounced snacks within 1 h before activity
- During exercise, limit carbohydrate intake to ≤20 g at a time, guided by CGM trends

Supplementary Table 2 – Evaluation of quality of life (Audit of Diabetes-Dependent Quality of Life questionnaire, ADDQoL), treatment satisfaction (Diabetes Treatment Satisfaction Questionnaire, DTSQs) and fear of hypoglycemia (Hypoglycemia Fear Survey II, HFS II) in participants, overall and according to AID system

|  | Overall | Medtronic | Tandem | Ypsomed |
| --- | --- | --- | --- | --- |
| Score evaluated | N = 86 | N = 39 | N = 25 | N = 22 |
| General quality of life score, from -3 (lower QoL) to +3 (better QoL) |  |  |  |  |
| Mean ± SD | 1.3 ± 0.7 | 1.4 ± 0.7 | 1.4 ± 0.6 | 1.1 ± 0.7 |
| Range | 0.0 - 3.0 | 0.0 - 3.0 | 0.0 - 2.0 | 0.0 - 2.0 |
| General quality of life score if the participant did not have diabetes, from -3 (much better) to +1 (worse) |  |  |  |  |
| Mean ± SD | -1.4 ± 1.1 | -1.4 ± 1.2 | -1.7 ± 0.9 | -1.3 ± 0.9 |
| Range | -3.0 - 1.0 | -3.0 - 1.0 | -3.0 - 0.0 | -3.0 - 1.0 |
| Weighted ADDQoL score, from -9 (maximum negative impact of diabetes) to +3 (maximum positive impact of diabetes) |  |  |  |  |
| Mean ± SD | -1.8 ± 1.4 | -1.7 ± 1.5 | -1.8 ± 1.2 | -1.8 ± 1.4 |
| Range | -6.3 - 0.0 | -6.1 - 0.0 | -4.7 - -0.4 | -6.3 - -0.1 |
| Overall treatment satisfaction score, from 0 to 36 (highest level of satisfaction) |  |  |  |  |
| Mean ± SD | 30.1 ± 3.7 | 30.2 ± 3.6 | 30.1 ± 3.9 | 29.7 ±3 .6 |
| Range | 22.0 - 36.0 | 22.0 - 36.0 | 23.0 - 36.0 | 23.0 - 36.0 |
| Behavior HFS II score, from 0 (never) to 4 (almost always) |  |  |  |  |
| Mean ± SD | 6.8 ± 3.2 | 6.5 ± 3.6 | 7.8 ± 3.3 | 6.1 ± 2.1 |
| Range | 0.0 / 16.0 | 0.0 / 16.0 | 0.0 / 15.0 | 1.0 / 12.0 |
| Worry HFS II score, from 0 (never) to 4 (almost always) |  |  |  |  |
| Mean ± SD | 5.3 (± 3.2) | 5.2 (±2.9) | 5.6 (±3.6) | 5.3 (±3.3) |
| Range | 0.0 - 15.0 | 0.0 - 15.0 | 0.0 - 14.0 | 0.0 - 14.0 |

Supplementary Table 3 – Participant-level analysis of hypoglycemia occurrence during at least one physical activity session, overall and by AID system.

|  | Overall | Medtronic | Tandem | Ypsomed |
| --- | --- | --- | --- | --- |
| Hypoglycemia occurrence | n = 86 | n = 39 | n = 25 | n = 22 |
| Participants with available data, n (%) | 73 (85) | 36 (92) | 21 (84) | 16 (73) |
| No hypoglycemia occurrence, n (%) | 16 (22) | 8 (22) | 4 (19) | 4 (25) |
| Hypoglycemia occurrence, n (%) | 57 (78) | 28 (78) | 17 (81) | 12 (75) |
| Proportion of PA sessions with hypoglycemia (<70mg/dL) |  |  |  |  |
| Mean ± SD | 27.3 ± 8.9 | 23.8 ± 16.5 | 27.2 ± 23.8 | 35.6 ± 14.6 |
| Range | 3.9 - 100.0 | 3.9 - 62.5 | 6.3 - 100.0 | 10.0 - 60.0 |
| Level 2 hypoglycemia occurrence, n (%) | 28 (38) | 12 (33) | 8 (38) | 8 (50) |
| Proportion of PA sessions with level 2 hypoglycemia |  |  |  |  |
| Mean ± SD | 19.2 ±14.6 | 11.8 ±6.8 | 26.7 ±21.9 | 22.7 ±10.1 |
| Range | 3.6 - 77.8 | 3.6 - 25.0 | 6.3 - 77.8 | 9.1 - 40.0 |

Supplementary Table 4 – Session-level analysis of self-reported hypoglycemia characteristics, overall and by AID system.

|  | Overall | Medtronic | Tandem | Ypsomed |
| --- | --- | --- | --- | --- |
| Hypoglycemia characteristics | n = 954 | n = 514 | n = 270 | n = 170 |
| Session with self-reported hypoglycemia, n (%) | 191 (20) | 87 (17) | 57 (21) | 47 (28) |
| Session with level 1 self-reported hypoglycemia, n (%) | 125 (13) | 68 (13) | 30 (11) | 27 (16) |
| Session with level 2 self-reported hypoglycemia, n (%) | 66 (7) | 19 (4) | 27 (10) | 20 (12) |
| Time of hypoglycemia occurrence, n (%) |  |  |  |  |
| Only prior to PA | 22 (12) | 13 (15) | 3 (5) | 6 (13) |
| Only during PA | 84 (44) | 41 (47) | 25 (44) | 18 (38) |
| Only after PA | 63 (33) | 24 (28) | 18 (32) | 21 (45) |
| Before and during PA | 2 (1) | 1 (1) | 1 (2) | 0 (0) |
| Before and after PA | 2 (1) | 0 (0) | 1 (2) | 1 (2) |
| During and after PA | 18 (9) | 8 (9) | 9 (16) | 1 (2) |
| Symptomatic hypoglycemia, n (%) | 125 (65) | 51 (59) | 41 (72) | 33 (70) |
| Type of symptom, n (%) |  |  |  |  |
| Adrenergic | 28 (15) | 10 (12) | 8 (14) | 10 (21) |
| Neuroglycopenic | 7 (4) | 4 (5) | 1 (2) | 2 (4) |
| Adrenergic and neuroglycopenic | 81 (42) | 32 (37) | 29 (51) | 20 (43) |
| Temporary cessation of PA session, n (%) | 36 (19) | 17 (20) | 14 (25) | 5 (11) |
| Level 1 self-reported hypoglycemia | 21 (11) | 15 (17) | 3 (5) | 3 (6) |
| Level 2 self-reported hypoglycemia | 16 (8) | 2 (2) | 11 (19) | 3 (6) |
| Permanent cessation of the PA session, n (%) | 23 (12) | 11 (13) | 9 (16) | 3 (6) |
| Level 1 self-reported hypoglycemia | 19 (10) | 10 (12) | 6 (11) | 3 (6) |
| Level 2 self-reported hypoglycemia | 5 (3) | 2 (2) | 3 (5) | 0 (0) |

Supplementary table 5 – Results of the mixed-effects logistic regression model assessing the impact of deviations from recommendations on the occurrence of reported hypoglycemia (N=697 PA sessions, including 153 with reported hypoglycemia)

| Variable | OR | 95% CI [OR] | p-value |
| --- | --- | --- | --- |
| Activation of exercise mode (Reference = activation ≥1h before PA) |  |  |  |
| Activation <1h before or during PA | 1.31 | [0.70 ; 2.46] | 0.687 |
| No activation | 1.15 | [0.55 ; 2.38] |  |
| Under-announcement of a meal within 2h before PA, without setting a temporary target | 0.64 | [0.17 ; 2.42] | 0.508 |
| Snack within 1h before PA, without bolus | 0.67 | [0.39 ; 1.16] | 0.152 |
| Snack >20 g consumed in one intake during PA | 1.15 | [0.55 ; 2.40] | 0.714 |
| Number of deviations before/during PA session (reference = 0) |  |  |  |
| 1 | 0.90 | [0.45 ; 1.79] | 0.891 |
| 2 | 0.77 | [0.35 ; 1.69] |  |
| 3-4 | 1.12 | [0.25 ; 5.05] |  |


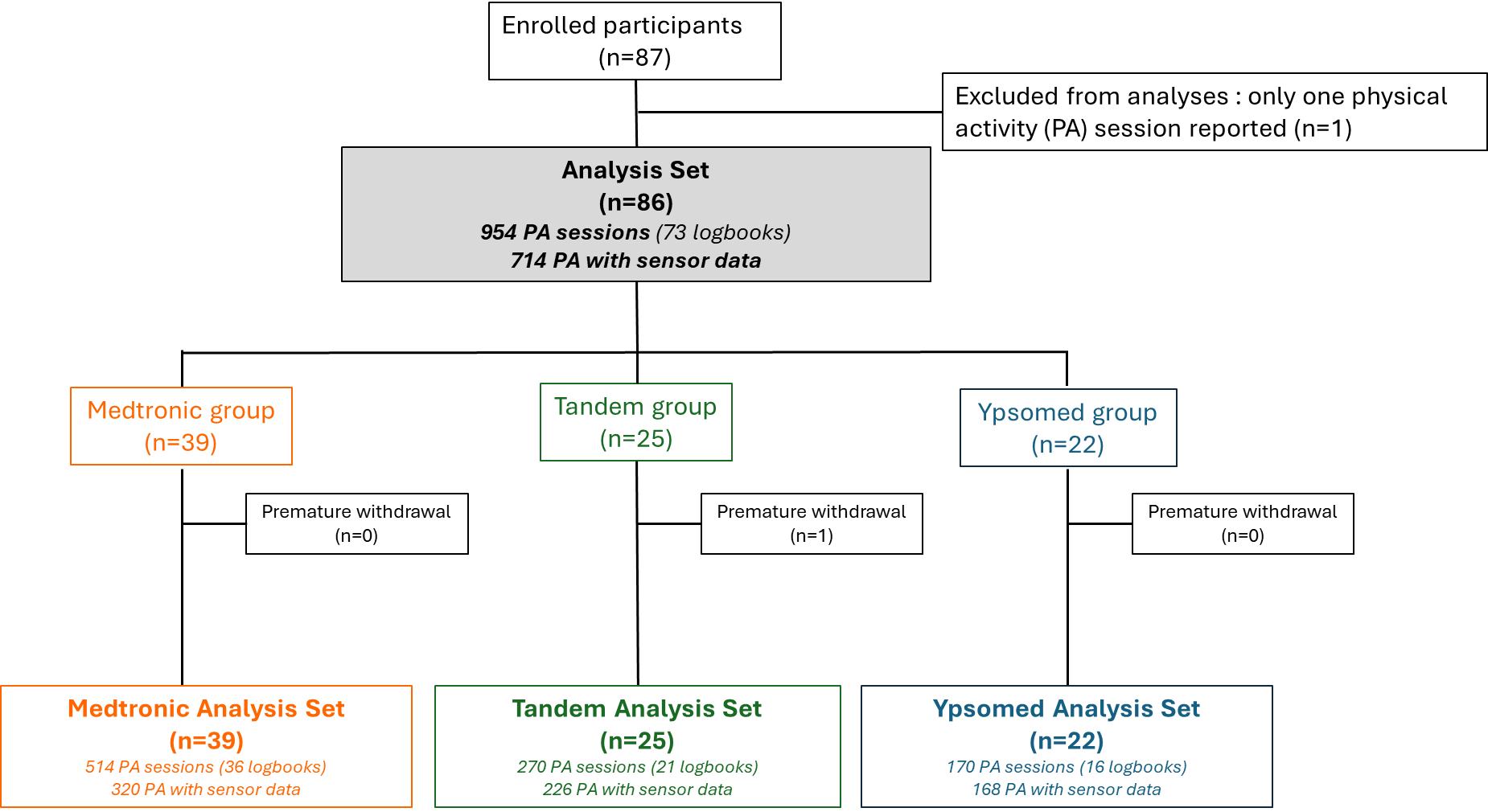


Supplementary Figure 1 – Participant flow chart

Supplementary – Method

**ADDQoL questionnaire:**

The Audit of Diabetes-Dependent Quality of Life (ADDQoL) is a questionnaire designed to assess how diabetes impacts various aspects of quality of life. It includes 3 quality of life scores:

The first score concerns the general quality of life. The patient selects their perceived quality of life from among seven options ranging from *“extremely poor”* to *“excellent.”* Responses are then interpreted by assigning a score from –3 (most negative response) to +3 (most positive response).

The second score is also a general item, asking the patient about his quality of life would be if they did not have diabetes. The patient chooses from five options ranging from *“much better”* to *“worse.”* These responses are scored from –3 to +1, with –3 reflecting a perception that life would be significantly better without diabetes, and +1 reflecting a perception that it would be worse.

The third score is a composite score derived from 19 items, each assessing the impact of diabetes on specific life domains: leisure activities, working life, mobility, holidays, physical ability, family life, social life, romantic relationships, sexual life, physical appearance, self-confidence, motivation, reactions of others, outlook on the future, financial situation, living conditions, dependence on others, freedom to eat, freedom to drink. For each domain, the patient first indicates whether the domain is applicable to their life. If so, he assesses how this aspect of life would be if they did not have diabetes, selecting from a range of responses from *“much better”* to *“worse.”* These are scored from –3 (maximum negative impact of diabetes) to +1 (positive impact of diabetes). The patient then assesses the personal importance of each domain, using a scale from 0 (not at all important) to 3 (very important).

A weighted impact score is then calculated for each applicable domain by multiplying the impact score by the importance rating:

Weighted score = Impact × Importance

Thus, each domain’s score can range from –9 (maximum negative impact) to +3 (maximum positive impact).

**DTSQ questionnaire:**

The overall treatment satisfaction score for the DTSQs ranges from 0 to 36 with a higher score corresponding to the higher level of satisfaction.

**HSF II questionnaire:**

The 'Behavior' scale of the HFS-II questionnaire is constructed by summing the scores from each of the 4 items in the 'Behavior' section of the questionnaire, with each item scored from 0 (Never) to 4 (Almost always).1.

The 'Worry' scale of the HFS-II questionnaire is constructed by summing the scores from each of the 4 items in the 'Worry' section of the questionnaire, with each item scored from 0 (Never) to 4 (Almost always).
